# Striking a balance: strategies for addressing single-use surgical equipment in infection prevention

**DOI:** 10.1017/ash.2025.186

**Published:** 2025-06-20

**Authors:** Steven S. Doerstling, Paige M. Fox, Jorge L. Salinas, Mindy M. Sampson

**Affiliations:** 1 Department of Medicine, Stanford University, Stanford, CA, USA; 2 Division of Plastic and Reconstructive Surgery, Department of Surgery, Stanford School of Medicine, Stanford, CA, USA; 3 Division of Infectious Diseases & Geographic Medicine, Department of Medicine, Stanford University, Stanford, CA, USA

## Abstract

Single-use surgical equipment is a standard strategy to reduce the risk of pathogen transmission in the operative room. However, this practice is associated with a great environmental impact. Reusable surgical tools represent an opportunity to reduce this impact, with many studies showing a 50% or greater reduction in carbon emissions by switching to reusable alternatives. While the safety of reusable equipment depends on strict sterilization protocols, the risk of infection is minimal when guidelines are followed. To advance sustainability in healthcare, we must balance infection prevention priorities, operational challenges, and the environmental considerations.

## Introduction

The environmental impact of healthcare is substantial. In the United States, the healthcare sector contributes an estimated 8%–10% of national greenhouse gas emissions,^1,2^ and each hospital bed can generate more than 10 kilograms of waste per day.^3^ Change is needed, and the most effective solutions must also ensure safety and maintain quality. One such opportunity is setting limits on the utilization of single-use surgical equipment (SSE).

SSE is widespread in operating rooms and procedure suites, and reducing its use could have a dramatic impact on healthcare’s environmental footprint.^4^ Despite the fact that SSE is commonly used, clinicians are concerned about the environmental impact of their work, and survey data suggest that a majority are willing to make changes that improve the sustainability of their practice.^5,6^ At the same time, it is important to understand the potential reasons why SSE remains a standard part of surgical practice. One key assumption may be that SSE is superior to reusable equipment for preventing the spread of infection between patients.^7,8^

**Figure 1.**
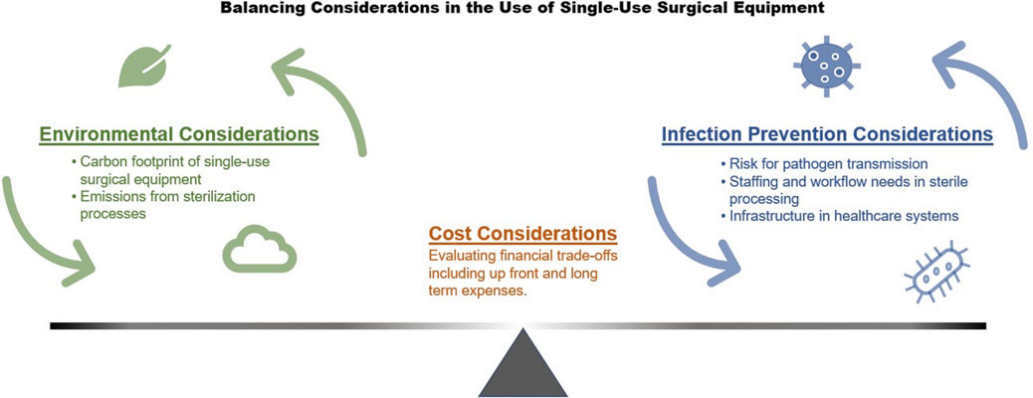
Conceptual framework illustrating the cost, environmental, and infection prevention considerations for reusable single-use surgical equipment.

This begs the question: Is SSE actually safer than reusable equipment, and if not, how might we balance the risks and benefits of its continued use considering the impacts on our environment and burden on healthcare systems (Figure 1)? This narrative review summarizes work on the implications and possible benefits of SSE, with an emphasis on identifying opportunities to limit its use.

## Environmental impact of single-use surgical equipment

A large body of evidence demonstrates that SSE has a significantly larger environmental impact compared to reusable alternatives. The majority of these studies are life cycle assessments, in which carbon emissions for a product are estimated. Almost universally, life cycle assessments of surgical equipment show that the carbon emissions from SSE can be reduced by more than 50% by switching to reusable tools.^9^

For example, a life cycle assessment of equipment for laparoscopic cholecystectomies found that the carbon footprint of hybrid equipment, which is predominantly reusable with only a small single-use component, was 76% lower than that of single-use equivalents.^10^ Similar findings have demonstrated a wide variety of reusable equipment can reduce the carbon footprint by more than 50%, including surgical scissors,^11^ robotic surgery ports,^12^ and anesthetic equipment.^13^

One limitation of life cycle analyses is that they often provide estimates for the relative reduction in carbon emissions when comparing different surgical approaches or equipment choices (e.g, single-use vs reusable). The comparisons are limited and may not fully capture how SSE impacts the absolute carbon emissions from a particular surgery. Some studies show that SSE accounts for a large share of the total environmental impact from surgical processes and equipment.^14^ An assessment of hysterectomy in the United States found that the production of single-use disposable materials was responsible for the majority of the environmental impact, when compared with other surgical processes including energy use, anesthetic gases, and the entire life cycle of reusable surgical equipment.^15^ Therefore, relative reductions in the environmental impact of SSE would translate into significant absolute reductions in the carbon footprint of surgery.

Another important limitation is that life cycle assessments of reusable surgical equipment do not always account for the resources needed for sterilization, which indirectly contribute to their environmental impact over time. Studies that included sterilization or decontamination as part of the life cycle have yielded mixed results on the net environmental benefit of reusable equipment compared with SSE in large part due to the type of energy used for sterilization.^10,13,16^

## Financial impact of single-use surgical equipment

Efforts to limit the use of SSE must contend with the financial consequences of changing the surgical supply chain. Studies as far back as the 1990s have generally demonstrated that reusable surgical equipment, especially laparoscopic instruments, cost less than single-use equivalents.^10,17,18^ For example, Royal Oak Hospital in Royal Oak, Michigan transitioned from disposable to reusable trocars, which generated an estimated annual savings of $275,000.^19^ However, it is also important to understand how these relative cost savings affect the absolute costs of surgery, but data are limited. Considering the significant differences in financing and supply chain contracts among healthcare systems, the financial impact of switching from SSE to reusable equipment on total surgical costs is difficult to generalize across hospitals.

Reusable surgical equipment requires sterilization, which represents an ongoing per-use cost that is not universally included in cost-effectiveness analyses.^20^ The maintenance of sterile processing infrastructure is especially challenging in low- and middle-income countries, where resource constraints are commonly cited as a barrier to maintaining World Health Organization (WHO) standards for sterile processing.^21,22^

Another contributor to the costs of reusable equipment is the need to sterilize all equipment from the operating room, whether it was used or not. One study of gynecologic surgeries found that less than one quarter of available instruments were used in the procedure, resulting in unnecessary sterilization costs.^23^

## Single-use versus reusable surgical equipment for infection prevention

The safety of reusable surgical equipment depends on strict adherence to international disinfection and sterilization guidelines. These guidelines are based on the Spaulding classification, which classifies instruments into three categories according to the risk of infection involved in their use, and then provides a rational approach to disinfection and sterilization based on the risk.^24^ Surgical instruments are classified as critical items, which enter sterile tissue or the vascular system and have the highest risk of transmitting infection. Critical items should undergo sterilization, usually with steam or other gases, with the goal of destroying all microorganisms, including bacterial spores. Semicritical items contact mucous membranes or nonintact skin and include instruments such as anesthesia equipment and some endoscopes. These items should undergo high-level disinfection with chemical disinfectants.^24^ For critical and semicritical items alike, it is also important to perform meticulous manual cleaning beforehand, as many of these devices have features such as narrow lumens or hinges that can make it difficult to eradicate microorganisms.

To evaluate the risk for transmission of infections, there are two key considerations: (1) the transmission of bacterial pathogens, such as gram-negative organisms, and (2) the transmission of blood-borne pathogens, such as HIV or viral hepatitis. While some may perceive SSE as being more effective than reusable equipment at preventing the spread of infection between patients, there is no high-quality evidence to support this claim.^25^ Additionally, there is evidence demonstrating that guideline-directed sterilization procedures are effective at preventing infection. A recent review found that the risk of acquiring a surgical site infection from reusable surgical instruments that underwent a validated sterilization cycle is “essentially zero.”^26^ Additionally, the transmission of bloodborne pathogens from improper sterilization is considered an exceedingly rare event in the United States, with only a few cases reported.^24^ Retrospective analysis of infections due to improper disinfection reveals that incidents are rare when sterilization protocols are correctly followed.^27^

Surgical site infections occur in 0.5 to 3% of patients undergoing surgery.^28^ While we should work to reduce the incidence of these infections, the low event rate poses challenges for designing randomized trials to reduce surgical site infections, which would require extremely high patient numbers to demonstrate even a modest benefit.^7^ Despite this challenge, a head-to-head randomized trial comparing SSE and reusable equipment for infection prevention is needed to confirm the findings from observational studies. In the meantime, available evidence suggests that properly sterilized reusable surgical equipment does not, on balance, pose a higher infectious risk compared with SSE.^24^

One important event in the recent history of infection prevention was the transmission of multidrug-resistant *E. coli* associated with exposure to duodenoscopes. In 2013, a cluster of 9 patients was identified as harboring New Delhi metallo-beta-lactamase-producing *E. coli*, which was linked to having undergone gastrointestinal endoscopy at a single hospital in northeastern Illinois.^29^ The causative organism was cultured from the endoscope used in 5 of the case patients, and thus established as the source of transmission. Specifically, the bacteria were recovered from the endoscope’s elevator channel, which is a small and complex component through which several different instruments can be passed. Further investigation showed that there were no lapses in the endoscope reprocessing procedures, malfunction of the automated endoscope reprocessor, or damage of the culprit endoscopes.^30^

Previous infectious outbreaks associated with endoscopes had been traced to cleaning or disinfection failures, but the 2013 outbreak occurred despite adhering to reprocessing guidelines, thus calling the adequacy of these guidelines into question.^30^ This event, in addition to subsequent outbreaks of multidrug-resistant bacteria associated with duodenoscopes, prompted the Food and Drug Administration (FDA) to propose strategies for preventing the spread of infection, including strengthening procedures to ensure manual cleaning prior to disinfection or sterilization, and requesting manufacturers to redesign duodenoscopes for easier cleaning.^31^ Currently, duodenoscope manufacturers have developed newer models with disposable components as well as fully disposable models, and both are supported by the FDA.

The challenge of preventing infection associated with endoscopes highlights important lessons to ensure the continued safety of reusable surgical equipment. First, updated guidelines and strong regulatory oversight are critical guardrails as surgical practice evolves. Second, implementing automated cleaning processes prior to sterilization reduces lapses or deficiencies related to human factors. Third, reusable surgical devices should be designed with cleaning and sterilization in mind. Fourth, standardized reporting procedures should be deployed so that clinicians and health systems are encouraged to communicate breakthrough infections, which is essential for accurate event rate monitoring and mobilizing regulatory resources to address reversible causes.

## Strategies to limit the use of single-use surgical equipment and improve surgical sustainability

### Education on the cost and environmental impact of SSE

Providing education on the environmental costs of SSE and the safety of reusable alternatives could influence institutional purchasing patterns. Surgeons, especially those in independent practice, may be receptive to the potential financial savings of reusable surgical equipment. A 2015 study found that educating surgeons on the costs of disposable equipment resulted in a 10% reduction in the supply costs of laparoscopic cholecystectomy over one year.^32^ In a survey of surgeons in the UK and Ireland, 57% of respondents cited cost as a perceived barrier to making changes toward environmental sustainability.^6^ Evidence-based education on the potential cost savings could further influence surgeons to make a sustainable change.

### Supplies and instrument use in the operating room

Surgical instruments and supplies should be available in a timely manner in the operating room, but there may be opportunities to streamline surgical instrument and supply selection to limit the use of disposable items. For example, one study found that unused or wasted supplies during neurosurgical procedures accounted for 13.1% of total surgical supply costs.^33^ Another study found that during laparoscopic hysterectomy, there was often redundancy in disposable and reusable instruments, and that avoiding redundancy could reduce greenhouse gas emissions by nearly 50%.^34^ Studies indicate that reducing the number of reusable instruments per tray can significantly reduce cleaning and reprocessing costs by reducing sterilization throughput.^35,36^ Efforts to optimize disposable supplies and instrument trays require coordinated efforts between surgeons, scrub technicians, operating room nurses, sterile processing, and supply chain.

### Investing in sterilization workforce and infrastructure

Transitioning from single-use to reusable equipment requires sufficient capacity in a healthcare system’s sterile processing department. These departments rely on a combination of technician-performed manual labor and automated cleaning and sterilization technologies to maintain the surgical instrument supply chain. Many institutions face staffing shortages in their sterile processing departments.^37^ The training requirements for sterile processing technicians is not standardized, and some positions require completion of a formal training program, which can take months. Subsidizing sterile processing technician training programs could lower the barrier to entry and expand the workforce. Simultaneously, hospitals could expand the throughput of their sterile processing departments by investing in automated cleaning technologies. One example is automated endoscope reprocessors, which are machines that replace some of the manual steps in endoscope reprocessing and have been shown to be safe and efficient.^38^

### Reprocessing single-use equipment

In the United States, the decision to label surgical equipment as single-use or reusable is made by the manufacturer. In order to market equipment as reusable, the manufacturer must submit data to the FDA showing that the equipment can be cleaned and sterilized without impacting its function. However, this does not mean that equipment labeled as single-use cannot be reprocessed for use. In fact, the FDA provides clearance for some SSE to be reprocessed by third-party reprocessing establishments.^39^ In 2008, the United States Government Accountability Office investigated reprocessed SSE and found no data to suggest it presents an elevated infection risk.^40^ As expected, studies have shown that reprocessing SSE can reduce costs and estimated carbon footprint^9^. When possible, surgical equipment seeking FDA approval under a reusable label should be given priority and/or expedited review. In addition, companies that reprocess single-use equipment could be subsidized to do so. For example, reprocessing companies could receive direct tax incentives, or governments could offer contracts to offer reprocessing services for publicly funded healthcare facilities.

Outside of the United States, there is wide variation in regulations and practice regarding reprocessing single-use equipment. WHO guidance recommends that critical and semi-critical devices must be reprocessed only by a licensed reprocessor. Furthermore, there is emphasis on the need for effective decontamination before sterilization, with a specific recommendation that devices with small lumens (eg catheters, drains, and fine cannulae) should not be reprocessed or reused.^41^ The European Union (EU) Regulation 2017/745 (Medical Device Regulation) states that the reprocessing of single-use devices is permissible only if permitted by national law of member nations.^42^ In fact, many member nations do not authorize this practice, and those that do permit reprocessing of single-use items enforce different restrictions.^43^ This variation represents a unique opportunity to investigate which specific national policies are most effective for balancing infection prevention, environmental stewardship, and cost.

### Extended producer responsibility

Extended producer responsibility (EPR) is a concept that holds manufacturers responsible for a product’s entire life cycle, including endpoints like disposal or recycling. One example of EPR policy outside of health care is trade-in programs for cell phones. This could be replicated in the surgical equipment market by incentivizing surgical equipment manufacturers to accept used surgical equipment as a trade-in for new equipment. The manufacturers, rather than the hospitals, would be responsible for reprocessing or recycling. Some may argue that manufacturers are better equipped to oversee these steps in the product’s life cycle, as they are already familiar with the logistics of raw materials handling and transportation. Healthcare systems, on the other hand, do not have this expertise, and should instead be focused on healthcare delivery.

### Tax and reimbursement incentives for healthcare systems

The Centers for Medicare and Medicaid provide reimbursements for a significant amount of healthcare services in the United States. These reimbursements have been used as incentives for hospitals to comply with certain standards, such as reporting quality metrics and preventing healthcare-associated infections. They could deploy a similar strategy to incentivize sustainability in the operating room. Reimbursements for surgery could be scaled inversely to the proportion of single-use equipment used in the procedure. The degree of reimbursement penalty need not be strict, but even a modest incentive could enable better data collection on how often SSE and reusable alternatives are used.

### Guidelines and leadership

Ultimately, medicine strives to be an evidence-based enterprise. Guidelines from professional organizations carry significant weight and shape practice on a large scale. Although further studies are needed to build upon the literature reviewed here, national and international bodies could develop guidelines on the use of disposable versus reusable surgical equipment and emphasize areas for further research. A recent survey of surgeons in Europe found that although a majority (82%) were willing to make changes to their clinical practice to improve environmental sustainability, 70% of respondents perceived a lack of leadership as a barrier to improving sustainability, and 91% of respondents welcomed greater leadership from national bodies.^6^ Similar sentiments have been reported among ophthalmologists in New Zealand,^44^ obstetricians in the United States,^5^ and anesthesiologists in Australia^45^ and Singapore.^46^ Reusable surgical equipment often (but not always) provides environmental benefits, and with the support of official guidelines on the appropriate use of these tools, clinicians are likely to change their practice accordingly.

## Conclusions

Reducing our reliance on SSE presents an opportunity for healthcare systems to make timely and important commitments toward environmental sustainability while maintaining safety, quality, and cost-effectiveness. Available evidence suggests that reusable surgical tools, when properly sterilized, results in a significantly lower carbon footprint without an increased risk of infection. However, higher quality evidence is needed to support the transition away from single-use equipment as the default option in many healthcare settings. Looking forward, scaling up sterilization and reprocessing capacity will be an essential part of a more climate-friendly surgical supply chain. Healthcare stakeholders must strike a balance between their professional obligation to prevent infections and their societal obligation to be stewards of the environment, with cost considerations as an important fulcrum (Figure 1). Executing these changes will require bold action from surgeons, payors, and professional societies alike.
